# Clear Cell Odontogenic Carcinoma of the Mandible Harboring *EWSR1* Rearrangement: Report of a Massive Jaw Tumor and Review of Diagnostic Considerations

**DOI:** 10.1155/2021/5558019

**Published:** 2021-03-09

**Authors:** Adepitan A. Owosho, Chukwubuzor Okwuosa, Donald I. Obi, Robinson O. Okiti, Kurt F. Summersgill

**Affiliations:** ^1^Missouri School of Dentistry and Oral Health, A.T. Still University, Kirksville, Missouri, USA; ^2^University of Nigeria Teaching Hospital, Nsukka, Enugu, Nigeria; ^3^University College Hospital, Ibadan, Oyo, Nigeria; ^4^Department of Diagnostic Sciences, School of Dental Medicine, University of Pittsburgh, Pittsburgh, Pennsylvania, USA

## Abstract

Clear cell odontogenic carcinoma (CCOC) is a low-grade malignant neoplasm that affects the jaws. We report an 18 cm massive case of mandibular CCOC in a 43-year-old female. The tumor was composed of nests and cords of round to polygonal monomorphic clear cells separated by prominent stromal hyalinization. Immunohistochemically, the tumor cells showed focal cytokeratin 5/6 positivity and intracytoplasmic PAS-positive granules and were negative for S100 and after diastase treatment (PAS-D). Molecularly, this case was positive for *EWSR1* rearrangement by FISH. The following should be included in the histopathological differential diagnosis: hyalinizing clear cell carcinoma of the salivary gland, clear cell variant of central mucoepidermoid carcinoma, clear cell variant of calcifying epithelial odontogenic tumor, and metastatic renal cell carcinoma. CCOC is a rare entity, with only 79 cases reported in the mandible. This case highlights the propensity for CCOC to exhibit invasiveness, destructive nature, and facial disfigurement if left untreated.

## 1. Introduction

Clear cell odontogenic carcinoma (CCOC) is a rare malignant odontogenic tumor with less 120 cases reported in the jaws since it was first described by Hansen et al. in 1985 [1]. CCOC was previously known as clear cell odontogenic tumor or clear cell ameloblastoma, when it was classified as a benign neoplasm [1, 2]. However, due to its aggressive clinical characteristics such as invasive growth, regional lymph node involvement, local recurrence, and distant metastasis, the WHO reclassified it as a malignant tumor of odontogenic origin in 2005. CCOC has a preference for females in the 5th decade of life and for the mandible, with 73.8% of reported cases occurring in the mandible [3].

Morphologically, CCOC presents as an infiltrative tumor composed of nests, sheets, or cords of monomorphic round to polygonal clear epithelial cells separated by variable hyalinized stroma [4]. Immunophenotypically, they are positive for cytokeratins, p63, and PAS, are diastase-sensitive (demonstrating intracytoplasmic glycogen), and are negative for mucicarmine and myoepithelial markers such as S100, SMA, and calponin [4, 5]. Molecularly, CCOC has been shown to demonstrate Ewing sarcoma region 1 (*EWSR1*) rearrangement [5]. In this article, we report a massive case of CCOC of the mandible in a Nigerian patient, performing limited immunohistochemical stains (CK5/6 and S100), special stains (PAS and, PAS-D), and FISH molecular analysis for *EWSR1* rearrangement.

## 2. Case Report

**Figure 1:**
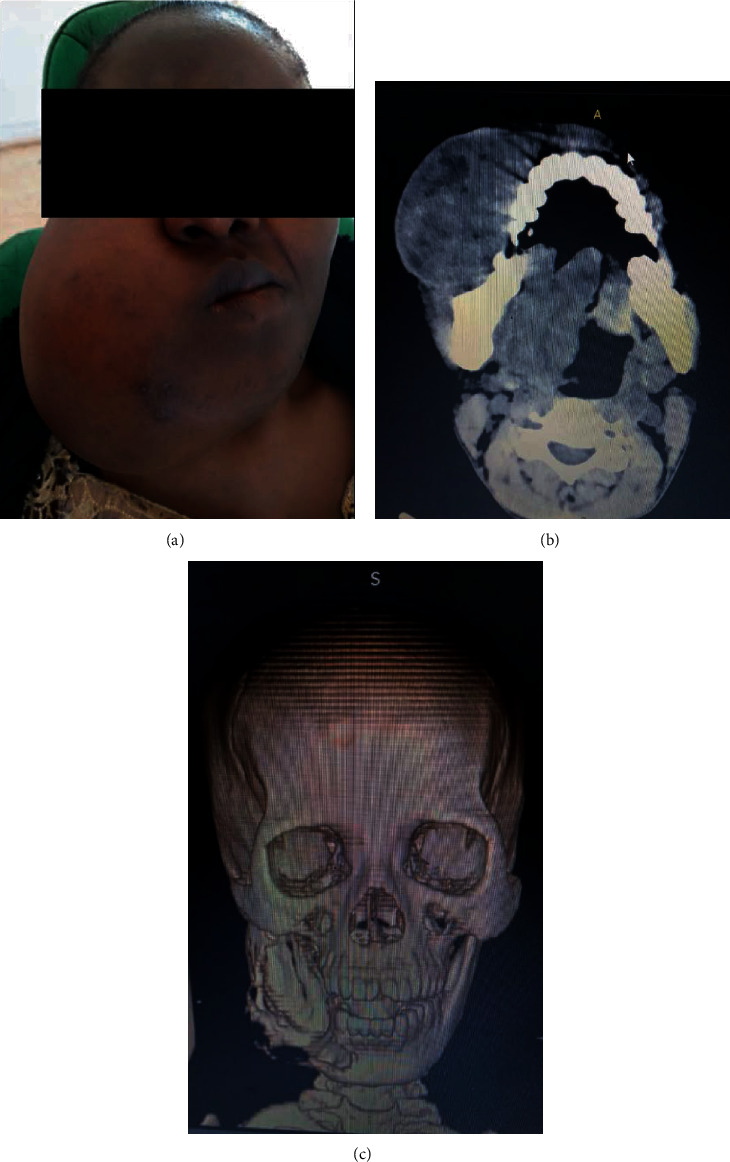
Clinical image of a 43-year-old female patient diagnosed with a *EWSR1*-rearranged clear cell odontogenic carcinoma of the mandible (a), CT scan axial view (b), and 3D-refomatted CT scan showing an osteolytic destructive mass in the mandible with thinning and perforation of the cortical border of the mandible (c).

A 43-year-old female patient presented in September 2019 with a 4-year history of a mandibular mass of 18 cm in diameter associated with buccolingual expansion of the mandible (Figure 1(a)) and regional lymph node involvement. Incisional biopsy had been performed 2 years prior, and an initial diagnosis of ameloblastoma was rendered. The mass was reported to have progressively increased in size for the past 2 years prior to presentation. Imaging revealed an osteolytic destructive mass in the mandible with thinning and perforation of the cortical border of the mandible (Figures 1(b) and 1(c)). A rebiopsy was performed.

**Figure 2:**
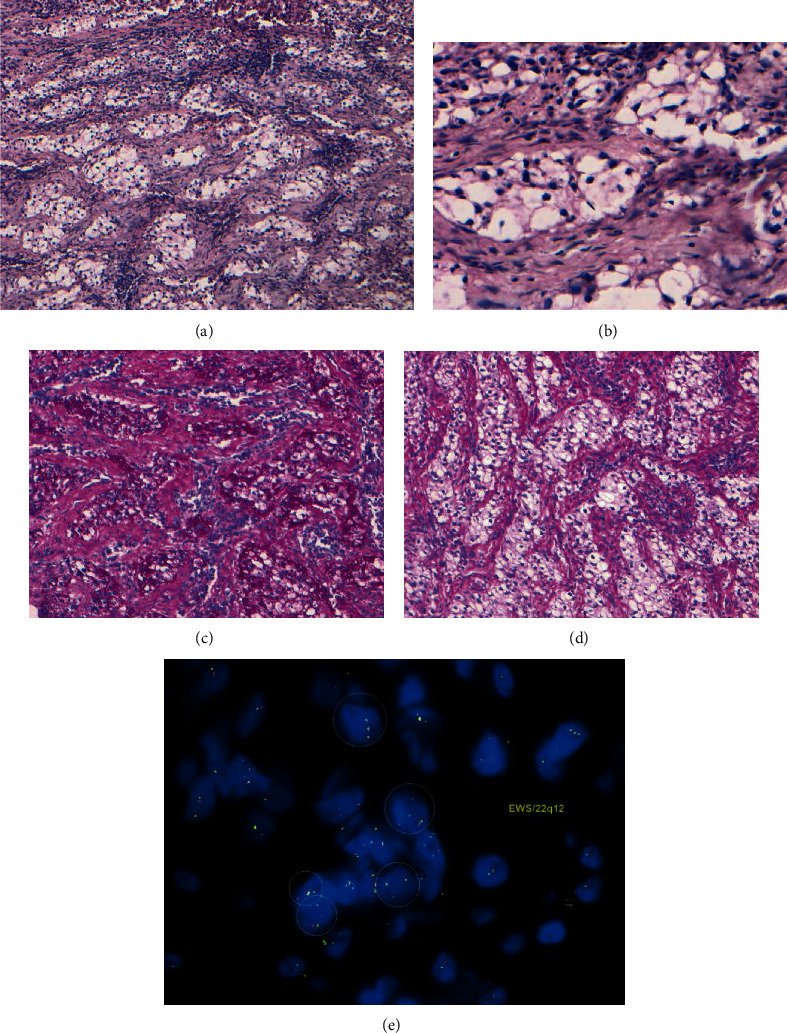
Histopathology of clear cell odontogenic carcinoma of the mandible in a 43-year-old female patient. (a) Morphology shows nests and cords of round to polygonal monomorphic clear epithelial cells with prominent stromal hyalinization containing lymphocytes (H&E 100x). (b) Higher magnification (H&E 400x). (c) Clear epithelial cells positive for PAS demonstrating intracytoplasmic granules (PAS 200x). (d) Clear epithelial cells are negative after PAS-D (200x). (e) Split of the green signal from the red signal by break-apart FISH demonstrates that the tumor harbors *EWSR1* rearrangement. In contrast, the uninvolved *EWSR1* allele shows a yellow signal (resulting from the fused red and green probes).

Morphologic features of the tumor were of nests and cords of round to polygonal monomorphic clear epithelial cells with prominent stromal hyalinization (Figures 2(a) and 2(b)). The tumor was poorly circumscribed and infiltrative with the presence of lymphocytes in the stroma. The tumor was positive for PAS but negative for PAS-D (demonstrating the presence of intracytoplasmic glycogen) (Figures 2(c) and 2(d)), showed focal expression for CK5/6, and was negative for S100 protein. FISH for *EWSR1* rearrangement was positive with 65% of translocated cells in 60 cells examined (Figure 2(e)). The cut-off for a positive *EWSR1* rearrangement is 12.5% of cells translocated.

**Figure 3:**
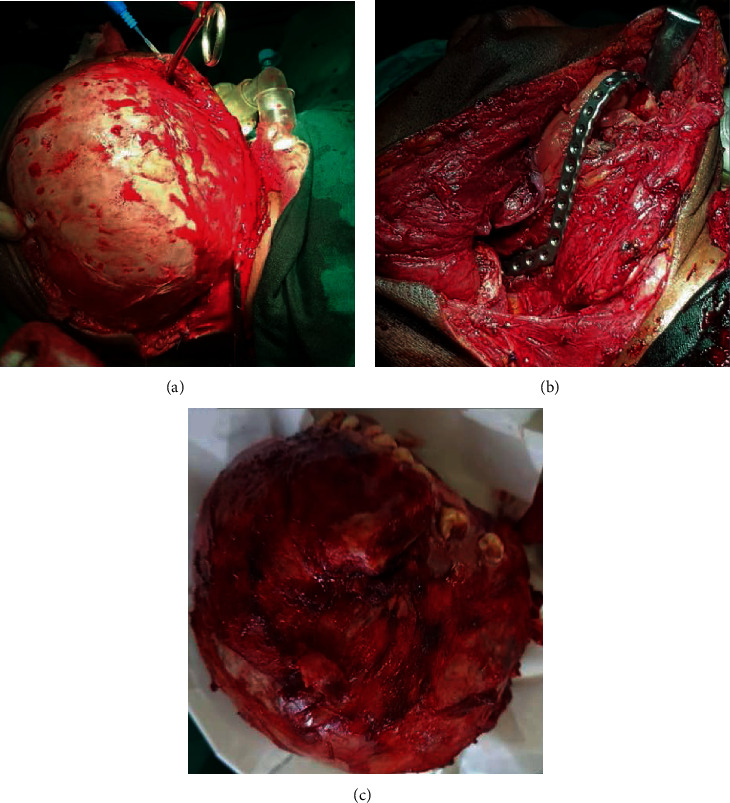
(a) Surgical exposure and resection of the EWSR1-rearranged clear cell odontogenic carcinoma of the mandible. (b) Reconstruction of the surgical defect with a mandibular reconstruction plate. (c) Massive resected *EWSR1*-rearranged clear cell odontogenic carcinoma of the mandible.

Two months after presentation, a subtotal mandibulectomy with neck dissection was performed for the resection of the CCOC (Figures 3(a)–3(c)). The patient is alive without disease and no recurrence at the last follow-up 15 months posttherapy.

## 3. Discussion

Clear cell odontogenic carcinoma (CCOC) is a rare malignant odontogenic neoplasm characterized by *EWSR1* gene rearrangement. Less than 120 cases have been reported in the literature. A recent review of the literature of 107 patients diagnosed with CCOC showed a predilection for females, the 5th decade of life, and the mandible [3]. In the same review, 13.6% (12/88) of CCOC patients manifested regional nodal involvement, and 11.2% (12/107) of CCOC patients died of disease [3]. In this report, we present a case of CCOC in a 43-year-old female with progressive growing mass, 18 cm in size in its largest diameter, located in the mandible associated with regional lymph node involvement and alive without disease and no recurrence 15 months posttherapy.

*EWSR1* rearrangement in CCOC was first described by Bilodeau et al.; the fusion partner identified was the activating transcription factor 1 gene (*ATF1*) on chromosome 12q13 [5]. An alternative fusion partner, *CREB1*, on chromosome 2q34 has been described [6]. *ATF1* and *CREB1* both belong to the cAMP response element-binding protein (CREB) family of transcription factors. *ATF1* remains the most common fusion partner to *EWSR1* identified in CCOC to date. Most cases of CCOC reported in the literature were described before *EWSR1* rearrangement was identified in the tumor. *EWSR1* gene rearrangement has been reported in numerous tumors, such as Ewing sarcoma, extraskeletal myxoid chondrosarcoma, clear cell sarcoma, desmoplastic small round cell tumor, myxoid liposarcoma, angiomatoid fibrous histiocytoma, primary pulmonary myxoid sarcoma, myoepithelial tumors of soft tissue, skin, and salivary glands, and hyalinizing clear cell carcinoma. *EWSR1-ATF1* translocation has been reported in other tumors, such as angiomatoid fibrous histiocytoma, hyalinizing clear cell carcinoma, and clear cell sarcoma [7–9]. Additionally, *EWSR1-CREB1* translocation has been reported in myxoid mesenchymal tumors, angiomatoid fibrous histiocytoma, primary pulmonary myxoid sarcoma, and gastrointestinal-clear cell sarcoma [10–13].

The differential diagnosis of CCOC includes hyalinizing clear cell carcinoma (HCCC) of the salivary gland, clear cell variant of central mucoepidermoid carcinoma (MEC), clear cell variant of calcifying epithelial odontogenic tumor (CEOT), and metastatic renal cell carcinoma. HCCC of the salivary gland is a rare low-grade malignant neoplasm that typically affects the minor salivary glands [14]. It has been argued that CCOC and HCCC are essentially the same analogous tumors with different locations [4, 5]. CCOC occurs centrally in the bone while HCCC occurs peripherally in the submucosa. CCOC and HCCC share similar morphologic, immunophenotypic, and molecular features [5].

MEC is the most common malignant salivary gland tumor that needs to be differentiated from CCOC, despite the presence of intercellular bridging of true epidermoid areas. MEC is composed of epidermoid, mucinous, and intermediate cells in variable proportion depending on its grade, forming solid and cystic patterns. MEC has been described in the jaw bone [15]. Clear cells have been described in MEC, and when predominant, they are called the clear cell variant of MEC. Immunohistochemical stains are not helpful in differentiating MEC from CCOC. However, PAS-D shows positive intracytoplasmic globules in MEC, which are negative in CCOC [4, 16]. MEC is negative for *EWSR1* rearrangement but may be positive for *CRTC1/CRTC3-MAML2* fusion [17].

CEOT is a rare invasive epithelial odontogenic tumor also known as Pindborg tumor. CEOT is composed of sheets, nests, and/or cords of polygonal eosinophilic epithelial cells with a prominent stroma [18]. These epithelial cells may present with clear cytoplasm, making it challenging to differentiate it from CCOC. However, cellular pleomorphism and the presence of amyloid and Liesegang calcified rings in the stroma that define CEOT are absent in CCOC. The eosinophilic amyloid material can be highlighted with Congo red stain, showing the classic apple-green birefringence with polarized light.

Metastasis of renal cell carcinoma to jaw should also be considered in the differential diagnosis of CCOC. These patients may have a history of a renal mass, or the metastasis to the oral cavity may be the first indication of a malignancy [19]. The metastatic renal cell carcinoma will be positive for PAX8, CD10, and CAIX in contrast to CCOC and negative for *EWSR1* rearrangement.

The case described here originated from the jaw, negative for PAS-D, and positive for PAS and *EWSR1* rearrangement, ruling out the aforementioned differential diagnoses. In conclusion, we report a rare form of jaw neoplasm “clear cell odontogenic carcinoma” with a propensity for invasiveness, destructive nature, and facial disfigurement if left untreated in a Nigerian patient harboring *EWSR1* rearrangement.

## Data Availability

No data were used to support this study.
